# Novel Insights into Staphylococcus aureus Deep Bone Infections: the Involvement of Osteocytes

**DOI:** 10.1128/mBio.00415-18

**Published:** 2018-04-24

**Authors:** Dongqing Yang, Asiri R. Wijenayaka, Lucian B. Solomon, Stephen M. Pederson, David M. Findlay, Stephen P. Kidd, Gerald J. Atkins

**Affiliations:** a Centre for Orthopaedic & Trauma Research, Faculty of Health Sciences, University of Adelaide, Adelaide, South Australia, Australia; b Bioinformatics Hub, School of Biological Sciences, University of Adelaide, Adelaide, South Australia, Australia; c Australian Centre for Antimicrobial Resistance Ecology, University of Adelaide, Adelaide, South Australia, Australia; d Research Centre for Infectious Disease, School of Biological Sciences, University of Adelaide, Adelaide, South Australia, Australia; University of Arkansas for Medical Sciences; Skirball Institute of Biomolecular Medicine, New York University Medical Center

**Keywords:** *Staphylococcus aureus*, bone infection, osteomyelitis, periprosthetic joint infection, small-colony variant, stress adaptation

## Abstract

Periprosthetic joint infection (PJI) is a potentially devastating complication of orthopedic joint replacement surgery. PJI with associated osteomyelitis is particularly problematic and difficult to cure. Whether viable osteocytes, the predominant cell type in mineralized bone tissue, have a role in these infections is not clear, although their involvement might contribute to the difficulty in detecting and clearing PJI. Here, using Staphylococcus aureus, the most common pathogen in PJI, we demonstrate intracellular infection of human-osteocyte-like cells *in vitro* and S. aureus adaptation by forming quasi-dormant small-colony variants (SCVs). Consistent patterns of host gene expression were observed between *in vitro*-infected osteocyte-like cultures, an *ex vivo* human bone infection model, and bone samples obtained from PJI patients. Finally, we confirm S. aureus infection of osteocytes in clinical cases of PJI. Our findings are consistent with osteocyte infection being a feature of human PJI and suggest that this cell type may provide a reservoir for silent or persistent infection. We suggest that elucidating the molecular/cellular mechanism(s) of osteocyte-bacterium interactions will contribute to better understanding of PJI and osteomyelitis, improved pathogen detection, and treatment.

## IMPORTANCE

Periprosthetic joint infections (PJIs) are increasing and are recognized as one of the most common modes of failure of joint replacements. Osteomyelitis arising from PJI is challenging to treat and difficult to cure and increases patient mortality 5-fold. Staphylococcus aureus is the most common pathogen causing PJI. PJI can have subtle symptoms and lie dormant or go undiagnosed for many years, suggesting persistent bacterial infection. Osteocytes, the major bone cell type, reside in bony caves and tunnels, the lacuno-canalicular system. We report here that S. aureus can infect and reside in human osteocytes without causing cell death both experimentally and in bone samples from patients with PJI. We demonstrate that osteocytes respond to infection by the differential regulation of a large number of genes. S. aureus adapts during intracellular infection of osteocytes by adopting the quasi-dormant small-colony variant (SCV) lifestyle, which might contribute to persistent or silent infection. Our findings shed new light on the etiology of PJI and osteomyelitis in general.

## OBSERVATION

Joint arthroplasty is a common and highly successful procedure; however, more than 1% of primary hip (1) and 1 to 3% of primary knee (2) arthroplasties fail due to periprosthetic joint infection (PJI). PJI carries a high risk of in-hospital and 5-year mortality (3, 4), and the cure rate after 2 years has been reported to be as low as 67% (5). These poor outcomes are due in part to incomplete knowledge of PJI disease etiology (6). In bone, osteoblasts (7) and osteoclasts (8) have been investigated in relation to bacterial infection. However, insufficient attention has been given to the role of the osteocyte in bone infection, despite this cell type constituting approximately 90 to 95% of cells in bone (9, 10). It has been reported that bacteria can reside in osteocytes *in vivo* (11, 12), and a recent study (13) demonstrated the ability of Staphylococcus aureus to invade the lacuno-canalicular spaces of cortical bone in an experimental osteomyelitis model, although infection of osteocytes *per se* was not reported in that study. The result of direct interaction between human osteocytes and bacteria remains to be investigated.

**FIG 1:**
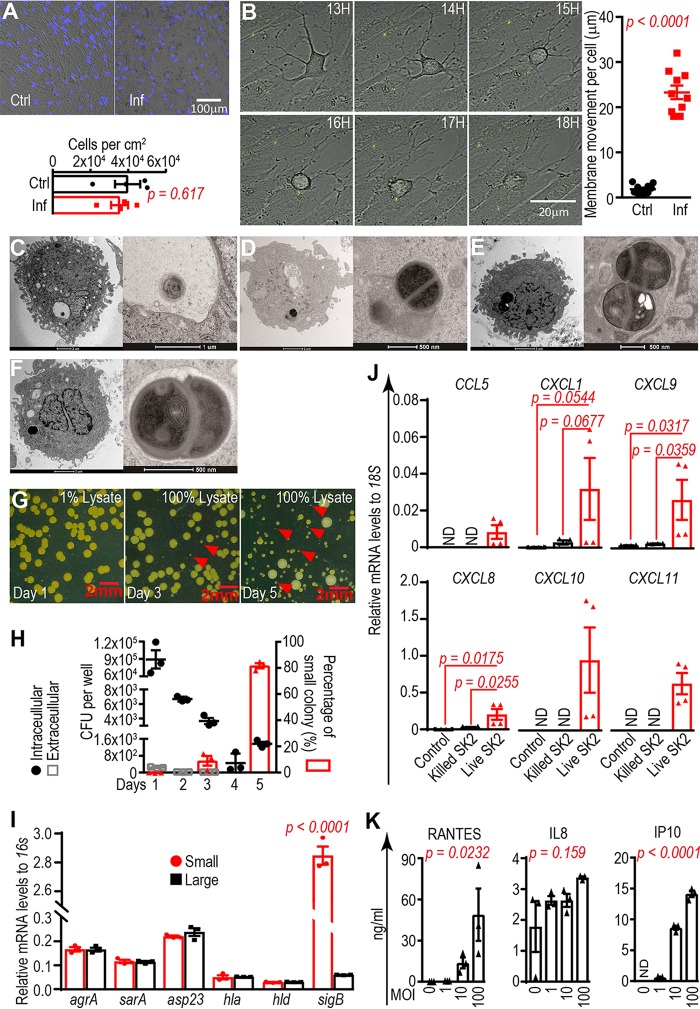
*In vitro* interaction between primary human-osteocyte-like cells and S. aureus. (A) Viability of osteocyte-like cells determined by DAPI staining of nuclei in the control (Ctrl) and infected (Inf) groups 30 days postinfection. (B) Time-lapse recording (13 to 18 h) of *in vitro*-differentiated human-osteocyte-like cell movement under S. aureus (strain RN6390; GFP labeled) infection. The sum of plasma membrane movements during the observation period was determined; data shown represent means ± standard errors of the means (SEM) for a pool of 10 cells from each of the Ctrl and Inf groups (*P* < 0.0001). TEM images of infected osteocyte-like cells showing dying S. aureus cells with compromised cell walls (C), proliferating S. aureus cells with evenly and completely divided cell walls (D), and proliferating S. aureus cells with unevenly and incompletely divided cell walls (E and F). (G) Recovered intracellular S. aureus on days 1, 3, and 5 featured with large and small (red arrowheads) sizes. (H) Quantification of intracellular and extracellular viable S. aureus CFU from days 1 to 5 (left *y* axis) and the percentage of small colonies recovered from host cells (right *y* axis) (graphs represent results from 3 independent biological replicates; values are means of results from the pool ± SEM). (I) Relative mRNA levels (normalized to 16S rRNA) of *agrA*, *sarA*, *asp23*, *hla*, *hld*, and *sigB* in recovered small and large colonies (values represent means from 3 independent biological replicates ± SEM). (J) Relative mRNA (normalized to 18S mRNA) levels of *CCL5*, *CXCL1*, *CXCL8*, *CXCL9*, *CXCL10*, and *CXCL11* at 24 h in osteocyte-like cells under control, killed-S. aureus WCH-SK2 and live-S. aureus WCH-SK2 treatments with an MOI of 100. Data shown are means of normalized expression ± SEM from 4 independent biological replicates. Comparisons (*t* tests) and *P* values are indicated. ND, nondetectable. (K) Production of the chemokines RANTES, IL-8, and IP10 by osteocyte-like cells after 24 h of exposure to S. aureus WCH-SK2 at MOIs of 0, 1, 10, and 100 (mean levels ± SEM from 3 independent biological replicates); the effect of each MOI on chemokine production was tested by one-way ANOVA, with relevant *P* values indicated.

Staphylococci, in particular S. aureus and the coagulase-negative species Staphylococcus epidermidis, have been identified as the most common causative pathogens in bone infection (14, 15). In this study, S. aureus was investigated because of its common association with bone infection and its well-characterized ability to exist in the quasi-dormant small-colony variant (SCV) lifestyle, which potentially contributes to a chronic disease state (16). The interaction between human osteocytes and S. aureus was investigated first by experimental infection using the WCH-SK2 strain (17), a methicillin-resistant S. aureus (MRSA) strain, at defined multiplicities of infection (MOIs). We utilized our well-characterized model of human-osteocyte-like cells generated by differentiation of human primary osteoblastic cultures to a mature, osteocyte-like state (18, 19). Day 28 differentiated osteocyte-like cells (Fig. S1) were cocultured with WCH-SK2 for 2 h, after which time extracellular bacteria were lysed using lysostaphin and washed to remove debris, leaving only internalized bacteria. There was no effect of infection on osteocyte-like cell viability, evidenced by the similar total cell numbers between the control and infected groups 30 days postinfection (Fig. 1A). Using live-cell imaging, uninfected osteocyte-like cells were observed to be relatively quiescent (see Movie S1 in the supplemental material), whereas cells exposed to green fluorescent protein (GFP)-expressing S. aureus (strain RN6390 [20]) were measurably active, with cell membranes and dendritic processes moving on average 25 µm nondirectionally in a 19-h time frame (Movie S2; Fig. 1B). Osteocyte-like cells were observed to have internalized WCH-SK2 cells; by 24 h postinfection, both dying bacteria with compromised cell walls (Fig. 1C) and actively proliferating S. aureus cells were observed in host cells (Fig. 1D to F). Among the proliferating S. aureus cells, distinctive phenotypes of dividing bacteria, including those with symmetrical and completely separated cell walls (Fig. 1D) and those with asymmetrical and incompletely separated cell walls (Fig. 1E and F), were observed. S. aureus cells with uneven and/or incomplete division were previously characterized as an adaptation to stress and, possibly, the initiation of SCV development. SCVs have a lower growth rate and lower virulence than normal cells but are able to persist inside host cells (16), avoiding immune and therapeutic antimicrobial interventions. Indeed, distinct bacterial colony types were grown from osteocyte-like lysates of cells of large (>1-mm) or small (<0.2-mm) sizes, the latter appearing as unpigmented (Fig. 1G). Overall, the total number of recovered CFU decreased from ~10^5^ per well at day 1 to ~1,500 per well at day 5 (Fig. 1H). At day 5, approximately 80% of the total recovered colonies were of the small, unpigmented phenotype (Fig. 1G and H). A comparison of the gene expression profiles of the small and large colonies (in pools of 10 colonies of each type) showed that the metabolism-related genes accessory gene regulator A (*agrA*), staphylococcal accessory regulator A (*sarA*), and alkaline shock protein 23 (*asp23*) and the bacterial-virulence-related genes alpha-hemolysin (*hla*) and delta-hemolysin (*hld*) were not different between colony types, whereas the stress adaptation-related gene sigma factor B (*sigB*) was found to be increased 50-fold in small colonies (Fig. 1I). These data are consistent with those of previous studies showing that the formation of SCV cell types is strongly associated with *sigB* expression level (21, 22). Our finding suggests that the development of SCVs was initiated following internalization by human-osteocyte-like cells. In response to S. aureus invasion, the expression of a number of chemokine genes, including the neutrophil chemoattractants *CCL5*, *CXCL1*, and *CXCL8* and chemokines related to T cell activation, *CXCL9*, *CXCL10*, and *CXCL11*, was strongly upregulated. The elevation of these chemokines was dependent on exposure to live S. aureus and did not occur in the presence of heat-killed cells (Fig. 1J). The protein production of *CCL5* (regulated upon activation, normal T cell expressed and secreted [RANTES]) and *CXCL10* (interferon gamma-induced protein 10 [IP10]) but not *CXCL8* (interleukin-8 [IL-8]) were confirmed to be secreted by exposed osteocyte-like cells in an MOI-dependent manner (Fig. 1K). A comprehensive transcription profile of the osteocyte-like cell response to S. aureus WCH-SK2 exposure *in vitro* was determined by microarray and bioinformatics analyses. These revealed a large set of 1,526 differentially expressed genes (Fig. S2A). Network analysis revealed numerous gene ontology (GO) term “communities” relating to a variety of host innate immune responses and cell metabolic activities (Fig. S2B).

10.1128/mBio.00415-18.1 FIG S1  Validation of the phenotype of osteocyte-like cells. (A) Presence of osteocytic markers, including *OCN*, *DMP1*, *SOST*, *RANKL*, and *FGF23*, in osteocyte-like cells differentiated for 28 days in comparison with the reference *18S* mRNA gene in a real-time PCR of the cDNA sample of the control group from the microarray experiment. (B) Our previous characterization of one of the donors of primary osteocyte-like cells used in the microarray experiment, demonstrating the fold change in mRNA levels of the osteocyte markers *DMP1* and *SOST* over a 4-week differentiation period. Data are presented normalized to day 0 values (means ± SEM from 3 independent biological replicates); the effect of differentiation time on each mRNA species was tested by one-way ANOVA, with relevant *P* values indicated. Download FIG S1, PDF file, 0.3 MB. Copyright © 2018 Yang et al. 2018 Yang et al. This content is distributed under the terms of the Creative Commons Attribution 4.0 International license.

10.1128/mBio.00415-18.2 FIG S2  Bioinformatics profiling of the osteocyte response to S. aureus. (A) Volcano plot showing all 1,526 differentially expressed (DE) genes and cutoff values for inclusion in the gene ontology (GO) enrichment analysis (the *x* axis represents the log_2_ values of mRNA fold changes [FC], and the *y* axis represents the −log_10_ values of significance [*P* values]). (B) The 202 enriched GO terms formed into communities based on the proportions of common DE genes between each pair of nodes. Each node represents a single GO term, with edges denoting the degree of connectedness between terms. Thicker edges correspond to a higher proportion of shared DE genes. Community hubs were defined as those with the most connections within each community and were used as community labels. The network layout and edge lengths were generated using a variation of the force-directed model. Download FIG S2, PDF file, 1.8 MB. Copyright © 2018 Yang et al. 2018 Yang et al. This content is distributed under the terms of the Creative Commons Attribution 4.0 International license.

10.1128/mBio.00415-18.5 MOVIE S1  Continuous imaging for 24 h of cell movements in the control group (related to Fig. 1B). Download MOVIE S1, AVI file, 16.8 MB. Copyright © 2018 Yang et al. 2018 Yang et al. This content is distributed under the terms of the Creative Commons Attribution 4.0 International license.

10.1128/mBio.00415-18.6 MOVIE S2  Continuous imaging for 24 h of cell movements in the infected group (related to Fig. 1B). Download MOVIE S2, AVI file, 16.8 MB. Copyright © 2018 Yang et al. 2018 Yang et al. This content is distributed under the terms of the Creative Commons Attribution 4.0 International license.

**FIG 2:**
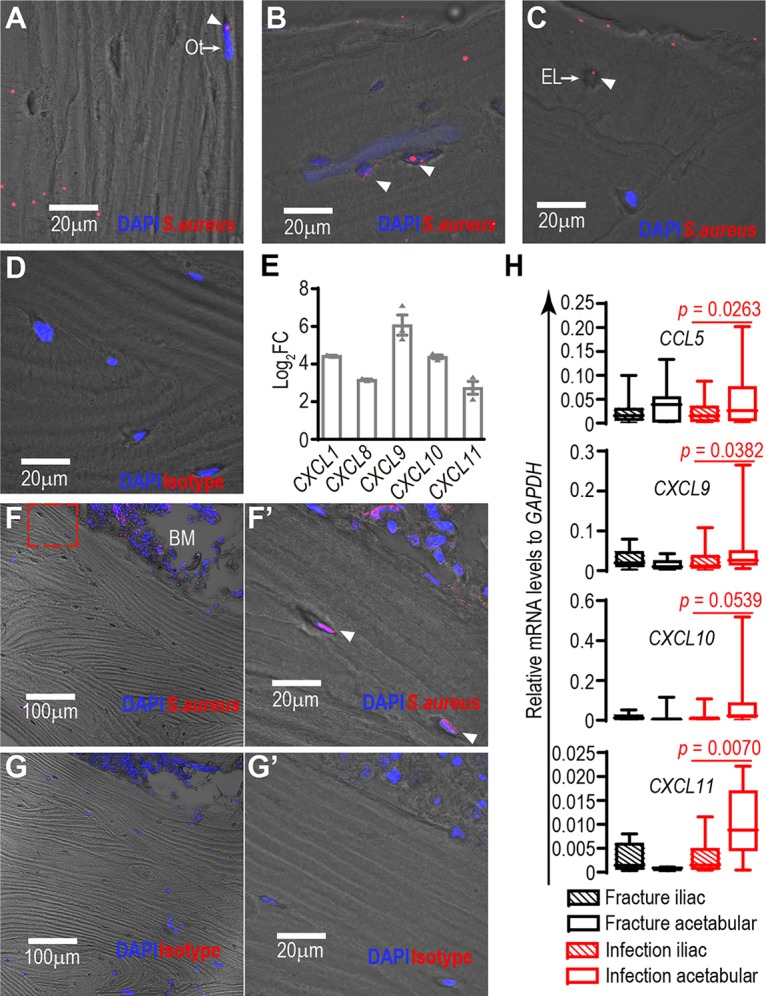
Interaction between osteocytes and S. aureus *ex vivo* and in clinical PJI specimens. (A and B) Presence of S. aureus (white arrowheads) in osteocyte (Ot)-occupied lacunae in bone tissue after *ex vivo* infection; (C) presence of S. aureus (white arrowhead) in empty lacunae (EL); (D) isotype-matched control immunostaining of infected bone tissue; (E) upregulation of mRNA levels of *CXCL1*, *CXCL8*, *CXCL9*, *CXCL10*, and *CXCL11* in infected bone samples in comparison to control samples (the *y* axis represents log_2_ values of mRNA fold change [FC] and mean normalized expression ± SEM from 3 independent biological replicates); (F) immunostaining targeting S. aureus (red fluorescence) in the acetabular bone of a PJI patient, with the box marked by a broken red line indicating the enlarged area in panel F′; (F′) high-magnification image of S. aureus staining of osteocytes/osteocyte lacunae (white arrowheads); (G) isotype-matched control immunostaining of PJI patient acetabular bone; (G′) high-magnification image of control staining of osteocytes/osteocyte lacunae; (H) boxplots (median with interquartile range) showing relative mRNA levels (normalized to that of the glyceraldehyde-3-phosphate dehydrogenase [GAPDH] gene) of *CCL5* (*P* = 0.026), *CXCL9* (*P* = 0.038), *CXCL10* (*P* = 0.054), and *CXCL11* (*P* = 0.0070) in bone specimens from the iliac wing and acetabulum of the PJI cohort (*n =* 23) and femoral fracture cohort (*n =* 13). *P* values indicate the comparisons between acetabulum bone and iliac wing bone from PJI patients. BM, bone marrow.

To investigate whether S. aureus is capable of penetrating human cancellous bone to contact osteocytes, fresh bone specimens were tested for susceptibility to bacterial infection *ex vivo*. Following a 12-h culture period, the presence of S. aureus in both occupied (Fig. 2A and B) and empty (Fig. 2C) osteocyte lacunae was observed by immunostaining. The specificity of antibody staining was validated by the use of a primary, isotype-matched control antibody (Fig. 2D). Consequently, the mRNA levels of *CXCL1*, *CLCX8*, *CXCL9*, *CXCL10*, and *CXCL11* were shown to be elevated following S. aureus invasion (Fig. 2E). The presence of intracellular S. aureus in osteocytes and the above-described host cell response were also confirmed in bone retrieved from PJI patients. Acetabular bone specimens from two patient cohorts, 24 with PJI (15 males and 9 females; age range, from 39 to 89 years) and 13 control patients with femoral neck fracture and no evidence of infection (3 males and 10 females; age range, from 22 to 87 years) were collected for examination. All members of the PJI cohort were S. aureus culture positive, as determined by routine diagnostic analysis of surgical biopsy specimens, and all were undergoing treatment by staged-revision total hip replacement surgery. A specimen from the iliac wing was also obtained from each patient to be able to compare the biopsy specimens with a skeletal site that was distal from the infected region and displayed no clinical signs of infection. All biopsy procedures were approved by the Human Research Ethics Committees of the Royal Adelaide Hospital and the University of Adelaide. Immunostaining (Fig. 2F), as well as intense staining of individual osteocyte lacunae and apparently viable osteocytes (Fig. 2F′), was carried out to examine specimens for the presence of S. aureus within bone tissue, and it identified S. aureus within the bone marrow. A three-dimensional reconstruction video of *in situ* confocal microscopy, scanning to a depth of 9 µm, was generated to confirm intracellular, cytoplasmic immunostaining of S. aureus (Movie S3). The specificity of staining was validated using isotype-matched antibody (Fig. 2G and G′). No positive staining was detected either in the corresponding iliac wing sample from the same patient or in acetabular samples from the femoral fracture cohort (Fig. S3A and S3B). Consistent with the presence of S. aureus in the PJI cohort bone, mRNA levels of a number of chemokines stimulated by infection, including *CCL5*, *CXCL9*, *CXCL10*, and *CXCL11* (Fig. 2H), were upregulated in bone specimens from the infected acetabulum site of PJI patients but not the uninfected iliac wing. Significantly elevated levels of these mRNA species were not observed in specimens from either acetabulum or iliac sites from the fracture cohort.

10.1128/mBio.00415-18.7 MOVIE S3  Three-dimensional reconstruction video of an infected osteocyte in Fig. 2F′, with the confocal microscope scanning to a depth of 9 µm. Download MOVIE S3, AVI file, 2.4 MB. Copyright © 2018 Yang et al. 2018 Yang et al. This content is distributed under the terms of the Creative Commons Attribution 4.0 International license.

10.1128/mBio.00415-18.3 FIG S3  Anti-S. aureus antibody staining. (A) Antibody staining targeting S. aureus in a PJI patient iliac wing bone; (B) antibody staining targeting S. aureus in a femoral fracture patient acetabular bone. Download FIG S3, PDF file, 1.7 MB. Copyright © 2018 Yang et al. 2018 Yang et al. This content is distributed under the terms of the Creative Commons Attribution 4.0 International license.

In summary, we have confirmed that S. aureus is capable of infecting human osteocytes both in *in vitro* cell cultures and in *ex vivo*-infected bone tissue. We also demonstrate the presence of S. aureus antigens in viable osteocytes present in regions of infected PJI patient bone. Our findings extend previous observations that undifferentiated osteoblasts can become infected by S. aureus (7) and that S. aureus can invade the lacuno-canalicular spaces of mouse cortical bone in an experimental infection model (13). Our *in vitro* and clinical findings demonstrate that osteocytes are capable of responding robustly to S. aureus invasion by producing immune mediators, consistent with previous findings with other nonprofessional antigen-presenting cell types, including osteoblasts (23). Some of the upregulated chemokines in this study, such as RANTES (24) and IP10 (25), were previously shown to have direct antimicrobial activity to clear extracellular bacteria. In our osteocyte-like cell model, whether the reduced live intracellular S. aureus cell number is associated with chemokine production, production of another mediator, or lysosomal digestion by the host cell or a combination of all three is an important question that needs to be addressed. This might potentially give rise to a novel therapeutic approach, stimulating osteocytes to clear the viable intracellular bacteria that are refractory to the effects of antibiotics. The bony location of the infected osteocyte may render them refractory to clearance by immune cells, and, as such, osteocytes may be an immune-privileged cell type. The osteocyte may therefore serve as a reservoir of bacteria for future reinfection, perhaps explaining the high prevalence of infections that become apparent only after long periods of time or recur following surgical/medical management.

Our findings also demonstrate the capacity for planktonic-phase S. aureus to rapidly switch to an SCV lifestyle upon interaction with osteocytes, indicative of an adaptation to stress from the host cell. A previous study identified the presence of S. aureus SCVs in fibroblasts in tissue from chronic PJI patients and suggested that infections with SCVs represented a problematic subset of cases (26). Our findings suggest that SCVs form upon interaction with osteocytes, and, as such, all S. aureus PJIs have the potential to become chronic if this occurs. SCVs have been demonstrated to be significantly less sensitive to most antibiotic treatments, and this mechanism of dormancy has been suggested to be a strategy for bacteria to survive, either in biofilm or inside host cells in chronic diseases (27). Other cell types, such as fibroblasts (26) and osteoclasts (8), have been proposed as reservoirs for S. aureus infection in PJIs; however, these are relatively short-lived cells. Osteoblast infection has also been proposed, and while the nature of this cell type is transient, the cell type is capable of differentiating into osteocytes, and infected osteoblasts have been shown to retain the ability to differentiate *in vitro* (7). Given that differentiated osteocytes are the most numerous and long-lived cell type encountered by bacteria in hard-bone tissue and demonstrate viability upon infection with intracellular S. aureus, osteocytes might be the ideal host cell type for S. aureus to survive in the long term. We propose that further investigation of the role of the osteocyte in PJI is warranted.

### Human-osteocyte-like cell model.

Isolation and maintenance of primary human osteoblasts were performed as previously established (18). Cells were then differentiated in alpha minimal essential medium (αMEM; Life Technologies, Inc., Grand Island, NY, USA) containing 5%, vol/vol, fetal calf serum (FCS; Thermo-Fisher Scientific, Scoresby, VIC, Australia), 1.8 mM potassium dihydrogen phosphate (Sigma-Aldrich, St. Louis, MO, USA), 100 µM ascorbate-2-phosphate (Sigma-Aldrich), and standard tissue culture l-glutamine–antibiotics (Life Technologies, Inc.). Cells were allowed to differentiate for 4 weeks in a humidified incubator in an atmosphere of 5% CO_2_–normoxia at 37°C.

### Bacterial strains.

The clinical isolate of S. aureus used, unless otherwise stated, was WCH-SK2 (17). S. aureus stain RN6390 engineered with green fluorescent protein (GFP) (20) was kindly provided by Clare Cooksley (Basil Hetzel Institute, SA, Australia). All bacterial-suspension cultures were maintained in Terrific broth (ThermoFisher Scientific, Carlsbad, CA, USA) at 37°C with agitation, and bacterial colonies were grown on Terrific broth with 1.2%, wt/vol, agar.

### Human bone biopsy specimens.

Bone specimens from two cohorts, namely, patients identified with S. aureus-related PJI following total hip replacement or a femoral fracture, were collected for immunohistochemistry and gene expression analyses. Samples from two different skeletal sites, the acetabulum and iliac wing, were included. Bone samples from femoral fracture patients were also utilized for *ex vivo* infection experiments. All the usage and operation procedures for collecting human bone biopsy specimens were approved by the Human Research Ethics Committees of the Royal Adelaide Hospital and the University of Adelaide.

### In vitro and ex vivo infection of osteocytes and bacterial colony recovery.

S. aureus WCH-SK2 actively growing in its log-phase stage was used for *in vitro* and *ex vivo* infection experiments, except for the live-cell imaging observation. A colorimetric method measuring light absorption of a bacterial suspension at 600 nm was employed to determine the number of CFU per volume for standardizing a multiplicity of infection (MOI). Bacterial suspensions were adjusted in phosphate-buffered saline (PBS) to achieve an MOI of 1, 10, or 100 for the purposes of testing the dosage-dependent effects of MOIs on the production of chemokine levels; otherwise, an MOI of 100 was used. Bacterial suspensions were then used to inoculate differentiated osteocyte-like cultures. After an initial infection period of 2 h, media were aspirated and replaced with antibiotic-free differentiation medium supplemented with 20 µg/ml lysostaphin (Sigma-Aldrich) to lyse remaining extracellular bacteria. An estimation of total host cell number 30 days postinfection in both control and infected cultures was carried out by counting the eukaryotic nuclei number using DAPI (4′,6-diamidino-2-phenylindole) staining and fluorescence microscopy (Olympus IX71 microscope) and by image analysis using ImageJ software (NIH, Bethesda, MD, USA). Osteocyte-like cultures were lysed in TRIzol reagent 24 h postinfection before performance of gene expression analyses as described below, and cell culture supernatants were collected for measuring secreted protein levels of RANTES (enzyme-linked immunosorbent assay [ELISA] kit; Caribbean Park, VIC, Australia), IL-8 (ELISA kit), and IP10 (DIP100; R&D Systems, Minneapolis, MN, USA) by ELISA. For *ex vivo* infection experiments, bone specimens from femoral fracture patients were dissected into 1-mm^3^ pieces and washed with PBS to remove the bone marrow fraction. Cleaned bone pieces were then cultured without bacteria as a control and with S. aureus adjusted to 10^3^ CFU per mm^3^ of bone volume as the infected group for 12 h. Specimens from both groups were then processed both for total RNA extraction using the TRIzol method and for immunostaining to detect S. aureus.

For the recovery of intracellular bacteria, osteocyte-like cultures were lysed in sterile water. Cell lysates were evenly distributed on Terrific broth/agar plates and cultured overnight at 37°C in 5% CO_2_. Agar plates were then kept at room temperature under aerobic conditions for 48 h to develop a gold pigmentation in resultant colonies, which is a typical feature of planktonic S. aureus. Following such growth development, colonies with a 0.2-mm diameter or less were counted as SCVs; both total colony counts and percentages of cells that were SCVs were recorded from each of the retrieval time points.

### Live-cell imaging of osteocyte-like cell infection.

For live-cell imaging, primary human osteoblasts were seeded in 8-well chamber slides (ibidi GmbH, Martinsried, Germany) and differentiated for 4 weeks, as described above. GFP-expressing S. aureus RN6390 was used to inoculate osteocyte-like cultures at an MOI of 10 for 2 h, followed by removal of extracellular bacteria by lysostaphin digestion. A Nikon TiE microscope with live-cell culturing components was utilized for imaging the interaction between host cells and bacteria over a 24-h time course. Osteocyte-like cells were visualized using the differential interference contrast (DIC) channel, and S. aureus cells were visualized using the GFP channel (excitation at 475 nm, emission collection between 520 and 560 nm). Images of identical regions within control and infected groups were taken every 5 min, and all serial images were stitched to generate video files for each treatment group. The video files in Movie S1 and S2 demonstrated the cell activities from 4 to 24 h. The movements of one infected cell from 13 to 18 h are depicted in Fig. 1B. Osteocyte-like cell plasma membrane movement was quantified from serial static images with a 30-min interval, using ImageJ software.

### TEM visualization of intracellular S. aureus.

Osteocyte-like cultures were infected with S. aureus WCH-SK2 in T75 flasks. Cells were harvested in pellet form 24 h postinfection and sequentially stabilized in fixative comprising 4%, wt/vol, paraformaldehyde–1.25%, wt/vol, glycolaldehyde in PBS (pH 7.2) and then in 2%, wt/vol, osmium tetroxide in water. Cell pellets were embedded in resin blocks, and 5-µm sections were taken for transmission electron microscopy (TEM; Philips CM200) imaging.

### Preparation of total RNA and reverse transcription (RT)-PCR analysis.

Total RNA samples were prepared from *ex vivo* bone tissue, *in vitro* osteocyte-like cultures, and bacterial colonies by using TRIzol reagent (Life Technologies, Inc.) as per the manufacturer’s instructions. For RNA isolation from bacteria, 30 min of lysostaphin (20 µg/ml) digestion at 37°C was performed prior to the addition of TRIzol (28). cDNA was synthesized using the iScript kit (Bio-Rad Laboratories, Hercules, USA). The mRNA expression of genes of interest was measured by real-time PCR using the RT^2^ SYBR Green quantitative PCR (qPCR) master mix (Qiagen, Limburg, the Netherlands), and relative expression was analyzed using the Δ*C*_*T*_ method (where *C*_*T*_ is threshold cycle). The efficiency of all primer pairs used (Table S1) was close to optimal, since a 10-fold dilution of the template resulted in an increase in *C*_*T*_ by 3.3-fold (data not shown).

10.1128/mBio.00415-18.4 TABLE S1  Primer sets used in this study. Download TABLE S1, PDF file, 0.04 MB. Copyright © 2018 Yang et al. 2018 Yang et al. This content is distributed under the terms of the Creative Commons Attribution 4.0 International license.

### Gene microarray and bioinformatics analysis.

For each sample, 400 ng total RNA was amplified using the Ambion whole-transcript (WT) expression kit (ThermoFisher), and the Affymetrix GeneChip WT terminus-labeling kit (ThermoFisher) was employed to produce biotin-labeled fragmented cDNA. Each labeled cDNA sample was hybridized to the Affymetrix human gene 2.0 ST array for 17 h at 45°C. Experimental triplicates of gene chip hybridization were carried out for both the control and infected groups. Microarray chips were washed and stained with a fluorescence-labeled antibody using a model 450 Affymetrix fluidics station. Gene chips were scanned using an Affymetrix 3000 7G scanner. Microarray analysis was performed using Partek Genomics Suite v6.5. The generated raw data as .Cel files were processed using the multiarray average (RMA) method (29). Comparisons between treatments were performed by analysis of variance (ANOVA), with Benjamini-Hochberg false-discovery rate (FDR) correction for multiple tests (30). Genes were considered significantly differentially expressed (DE) using an FDR-adjusted *P* value of <0.01 and an estimated log_2_ fold change (log_2_FC) beyond ±1, giving 1,526 unique Entrez Gene identifiers considered to be DE. The set of DE identifiers was tested for enrichment of Gene Ontology (GO) terms using the Fisher exact test and a set of background genes, defined as those included on the array but with an unadjusted *P* value of >0.05. GO terms with fewer than 4 steps back to the ontology roots were discarded, leaving 8,607 GO terms tested for enrichment. GO terms were considered enriched in the set of DE genes if they received a Bonferroni-adjusted *P* value of <0.05, yielding 202 enriched GO terms. For the purposes of visualization, GO terms were formed into communities using the common genes within each term (31), with the “hub” or most-connected GO terms labeled in Fig. S1B.

### Immunohistochemistry analyses on human bone biopsy specimens.

Bone specimens for immunohistochemistry analysis were stabilized and decalcified in TheraLin tissue fixative (Grace Bio-Labs, Bend, OR, USA) at room temperature for 3 days. Samples were then embedded in paraffin, and 10-µm sections from each of the samples were taken to perform immunohistochemistry staining. Antibody targeting S. aureus (AB37644; Abcam, Inc., Cambridge, MA, USA) was used at a 1:100 dilution for staining at 4°C for 1 h to detect the pathogen. Primary antibody signals were tagged by Alexa Fluor 647-conjugated rabbit anti-mouse IgG (H+L) secondary antibody (A-21239; Life Technologies, Inc.) for visualization. The specificity of antibody staining was validated by following the same staining procedures, using matched-isotype control antibody (14-4742-81; ThermoFisher) for primary detection. DAPI was employed to label nuclei of cells through all sections. All samples were imaged with an Olympus FV3000 confocal microscope. The underlying bone structure was visualized using the transmission detection function, which generates a gray-scale image of the tissue section surface.

### Statistical analyses.

One-tailed *t* tests with Welch’s correction were used for examining the differences in cell movements in Fig. 1B, mRNA levels of the chemokine gene panel in Fig. 1J, and gene expression in bone biopsy specimens between infection and iliac wing sites from the PJI cohort in Fig. 2H. One-tailed tests were used in the case of membrane movement because cells clearly changed from an inactive to an active state and in the case of host cell gene expression responses, because gene microarray analysis indicated that the genes examined were strongly induced. Two-tailed *t* tests with Welch’s correction were employed for examining the differences in total cell numbers in Fig. 1A and *sigB* mRNA levels in Fig. 1I, because no assumption was made for the behavior of either host cells or bacteria. One-way ANOVA tests were used for determining the effects of various MOIs on chemokine production levels in Fig. 1K. Exact *P* values for each test are shown on the respective figure, where applicable. Statistical analysis was not applicable for data sets containing nondetectable (ND) values.

### Accession number(s).

Microarray experiment raw files have been deposited in the server of ArrayExpress (https://www.ebi.ac.uk/arrayexpress/experiments/E-MTAB-6700/) and made available to the public.
