# Supplementary material for: Novel Insights into Staphylococcus aureus Deep Bone Infections: the Involvement of Osteocytes
Source: mBio. 2018 Apr 24;9(2):e00415-18. doi: 10.1128/mBio.00415-18 (PMC5915738; doi:10.1128/mBio.00415-18)
Supplement: FIG S1 [file mbo002183853sf1.pdf]

A

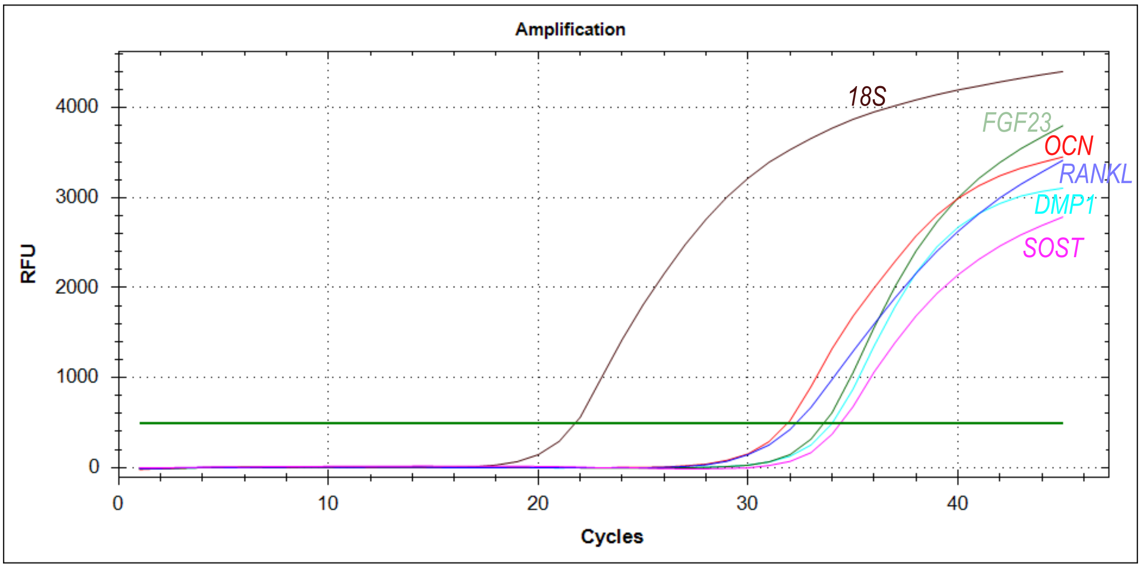

B

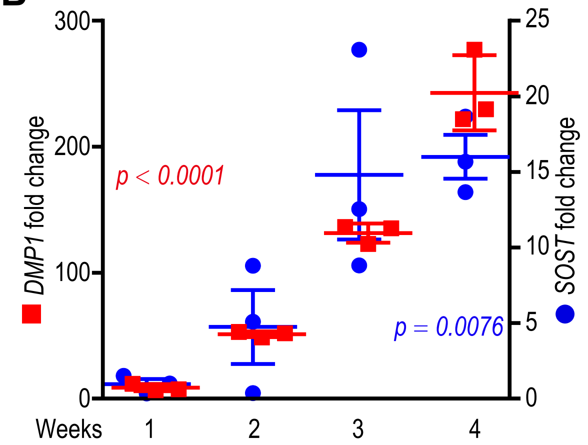

FIG S1 Validation of the phenotype of osteocyte-like cells. (A) The presence of osteocytic markers in osteocyte-like cells differentiated for 28 days, including *OCN*, *DMP1*, *SOST*, *RANKL* and *FGF23* in comparison to the reference gene 18S, in a real-time PCR reaction, in the cDNA sample of the Control group from the microarray experiment. (B) Our previous characterisation of one of the donors of primary osteocyte-like cells used in the microarray experiment demonstrating the fold-change in mRNA levels of osteocyte markers *DMP1* and *SOST* over a 4-week differentiation period. Data are presented normalised to day 0 values; means ± SEM of 3 independent biological replicates; the effect of differentiation time on each mRNA species was tested by One-way ANOVA, with relevant p values indicated.
