# Supplementary material for: Novel Insights into Staphylococcus aureus Deep Bone Infections: the Involvement of Osteocytes
Source: mBio. 2018 Apr 24;9(2):e00415-18. doi: 10.1128/mBio.00415-18 (PMC5915738; doi:10.1128/mBio.00415-18)
Supplement: FIG S2 [file mbo002183853sf2.pdf]

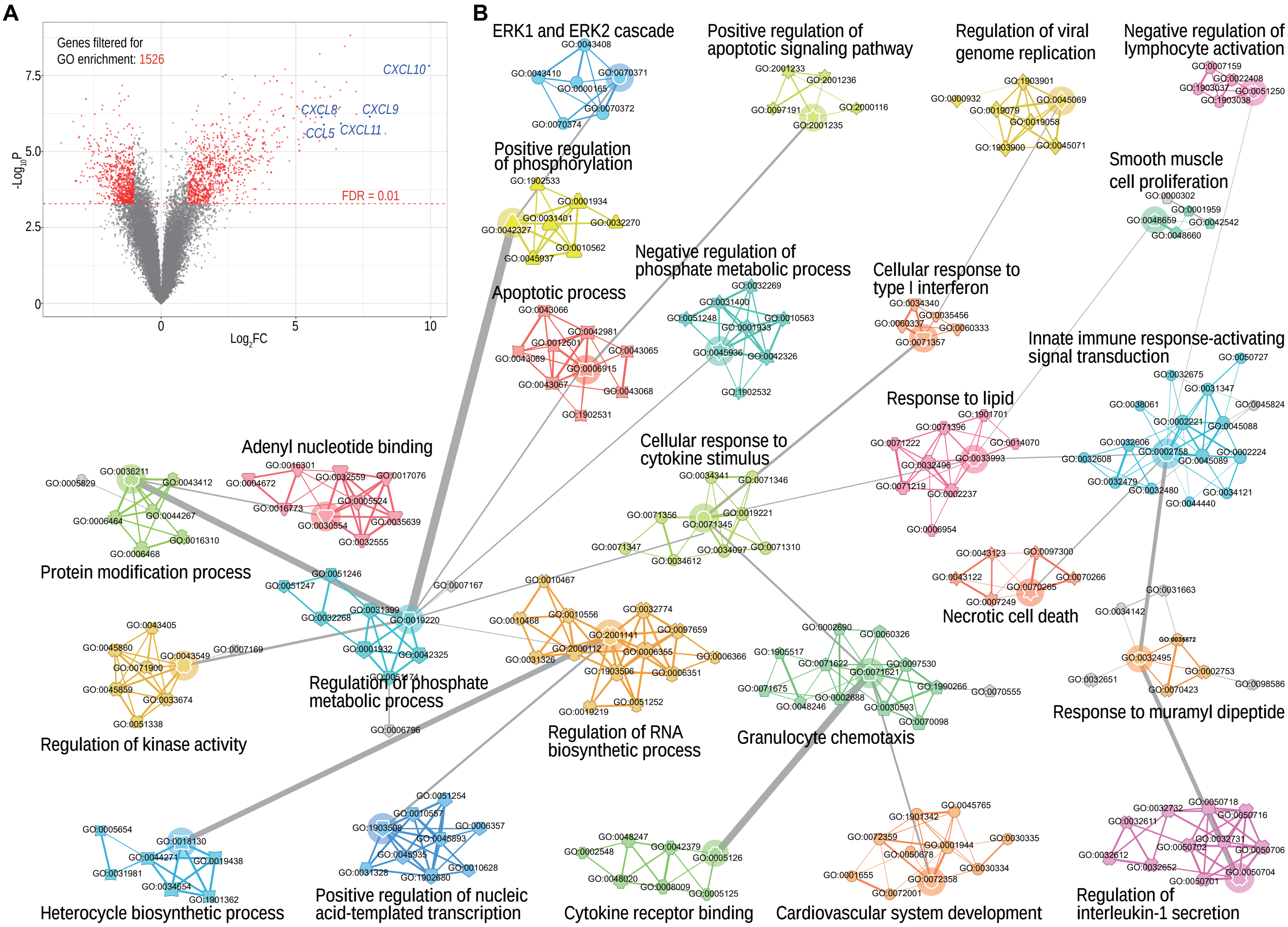

FIG S2 Bioinformatics profiling of the osteocyte response to *S. aureus*. (A) Volcano plot showing all 1526 differentially expressed (DE) genes and cut-off values for inclusion in the gene ontology (GO) enrichment analysis (x axis represents the Log2 values of mRNA fold changes (FC) and y axis represents the of -Log10 values of significance, p values). (B) The 202 enriched GO terms formed into communities based on the proportions of common DE genes between each pair of nodes. Each node represents a single GO term, with edges denoting the degree of connectedness between terms. Thicker edges correspond to a higher proportion of shared DE genes. Community hubs were defined as those with the most connections within each community and were used as community labels. The network layout and edge lengths were generated using a variation on the force directed model.
