# Supplementary material for: Novel Insights into Staphylococcus aureus Deep Bone Infections: the Involvement of Osteocytes
Source: mBio. 2018 Apr 24;9(2):e00415-18. doi: 10.1128/mBio.00415-18 (PMC5915738; doi:10.1128/mBio.00415-18)
Supplement: FIG S3 [file mbo002183853sf3.pdf]

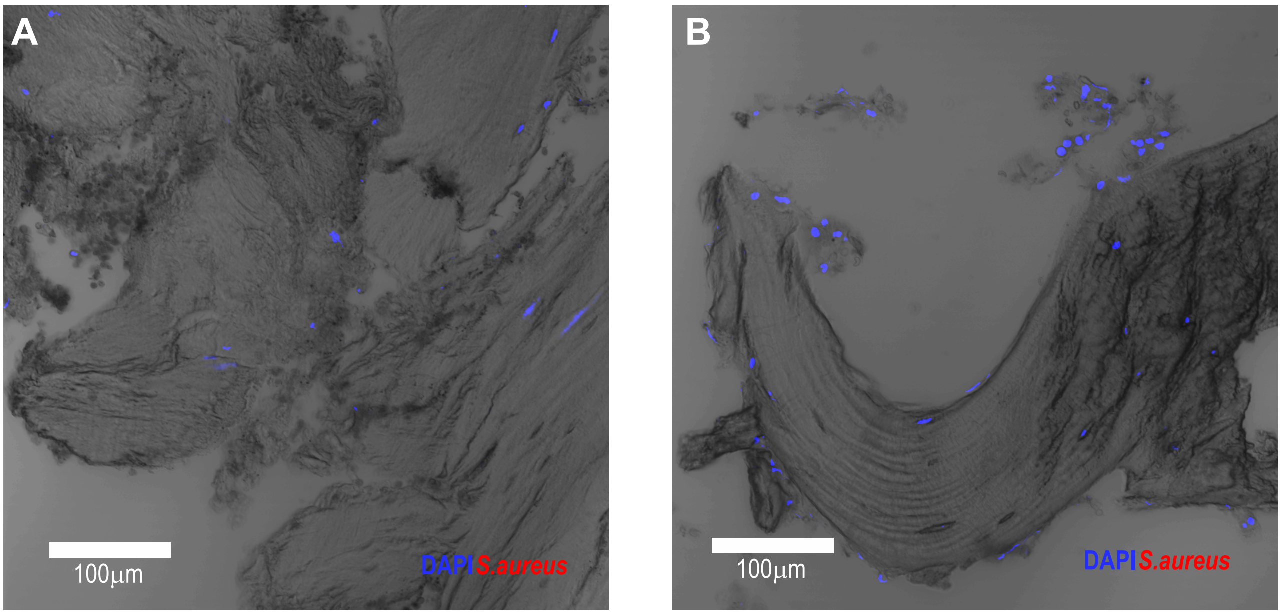

FIG S3 Anti-*S. aureus* antibody staining. (A) Antibody staining targeting *S. aureus* of PJI patient iliac wing bone. (B) Antibody staining targeting *S. aureus* of femoral fracture patient acetabular bone.
