# Supplementary material for: Novel Insights into Staphylococcus aureus Deep Bone Infections: the Involvement of Osteocytes
Source: mBio. 2018 Apr 24;9(2):e00415-18. doi: 10.1128/mBio.00415-18 (PMC5915738; doi:10.1128/mBio.00415-18)
Supplement: TABLE S1 [file mbo002183853st1.pdf]

TABLE S1 Primer sets used in this study

Gene name	Forward primer	Reverse primer	Amplicon size (bp)
16s *	gcgtgcctaacacatgcaa	gtcgccttggtaagccgtta	246
agrA (1)	aactgcacatacacgcttaca	ggcaatgagtctgtgagattt	145
sarA (2)	acatggcaattacaaaaatcaatgat	tctttctctttgttttcgctgatg	151
asp23 (2)	aaagcaaaacaagcatacgacaatc	agcgataccagcaatttttttcaac	149
hla (1)	caactgataaaaaagtaggctggaaagtgat	ctggtgaaaaccctgaagataatagag	201
hld (3)	taattaaggaaggagtgatttcaatg	tttttagtgaatttgttcactgtgtc	100
sigB *	ggggcaacaagatgaccatt	tgccgttctctgaagtcgtg	187
CCL5 *	gtgctccaacccagcagtc	catcctagctcatctccaaag	113
CXCL1 *	gcccaaaccgaagtcatagc	tgtcactgttcagcatcttttc	106
CXCL8 *	acatgacttccaagctggcc	ggtccagacagagctctcttcc	240
CXCL9 *	gagtgcaaggaaccccagtag	agggcttggggcaaattgtt	113
CXCL10 *	gtccacgtgttgagatcattgc	atcgattttgctcccctctg	151
CXCL11 *	tgctacagttgttcaaggcttcc	cactgcttttaccccagggc	76
DMP1 (4)	gatcagcatcctgctcatgtt	agccaaatgacccttccattc	125
FGF23 *	atgctggctttgtggtgatta	tctccgggtcgaaatagtgt	99
GAPDH (5)	acccagaagactgtggatgg	cagtgagcttcccgttcag	142
OCN (5)	atgagagccctcacactcctcg	gtcagccaactcgtcacagtcc	255
RANKL (5)	tcagccttttgctcatctcactat	ccaccccсgatcatggt	96
SOST (4)	accaccccctttgagaccaaag	ggtcacgtagcgggtgaagt	79
18S *	gcgttgattaagtccctgcc	cacctacggaaaccttgttacgac	181

* indicates the first use in this study

1. Tuchscherr L, Medina E, Hussain M, Volker W, Heitmann V, Niemann S, Holzinger D, Roth J, Proctor RA, Becker K, Peters G, Loffler B. 2011. Staphylococcus aureus phenotype switching: an effective bacterial strategy to escape host immune response and establish a chronic infection. EMBO Mol Med 3:129-41.

2. Tuchscherr L, Bischoff M, Lattar SM, Noto Llana M, Pfortner H, Niemann S, Geraci J, Van de Vyver H, Fraunholz MJ, Cheung AL, Herrmann M, Volker U, Sordelli DO, Peters G, Loffler B. 2015. Sigma Factor SigB Is Crucial to Mediate Staphylococcus aureus Adaptation during Chronic Infections. PLoS Pathog 11:e1004870.
3. Moisan H, Brouillette E, Jacob CL, Langlois-Begin P, Michaud S, Malouin F. 2006. Transcription of virulence factors in Staphylococcus aureus small-colony variants isolated from cystic fibrosis patients is influenced by SigB. J Bacteriol 188:64-76.
4. Atkins GJ, Welldon KJ, Holding CA, Haynes DR, Howie DW, Findlay DM. 2009. The induction of a catabolic phenotype in human primary osteoblasts and osteocytes by polyethylene particles. Biomaterials 30:3672-81.
5. Atkins GJ, Anderson PH, Findlay DM, Welldon KJ, Vincent C, Zannettino AC, O'Loughlin PD, Morris HA. 2007. Metabolism of vitamin D3 in human osteoblasts: evidence for autocrine and paracrine activities of 1 alpha,25-dihydroxyvitamin D3. Bone 40:1517-28.
